# Death and Transfiguration in Static *Staphylococcus epidermidis* Cultures

**DOI:** 10.1371/journal.pone.0100002

**Published:** 2014-06-25

**Authors:** Christoph Schaudinn, Paul Stoodley, Luanne Hall-Stoodley, Amita Gorur, Jonathan Remis, Siva Wu, Manfred Auer, Stefan Hertwig, Debbie Guerrero-Given, Fen Ze Hu, Garth D. Ehrlich, John William Costerton, Douglas H. Robinson, Paul Webster

**Affiliations:** 1 Centre for Biological Threats and Special Pathogens, Robert Koch Institute, Berlin, Germany; 2 Departments of Microbial Infection and Immunity and Orthopedics, Columbus, Ohio, United States of America; 3 Life Sciences Division, Lawrence Berkeley National Laboratory, Berkeley, California, United States of America; 4 Department of Biological Safety, Federal Institute for Risk Assessment, Berlin, Germany; 5 Electron Microscopy, Max Planck Florida Institute, Jupiter, Florida, United States of America; 6 Center for Genomic Sciences and Center for Advanced Microbial Processing, Institute for Molecular Medicine and Infectious Disease; Department of Microbiology and Immunology Drexel University College of Medicine, Philadelphia, Pennsylvania, United States of America; 7 deNovo Biologic LLC, Arlington, Virginia, United States of America; 8 Center for Electron Microscopy and Microanalysis, University of Southern California, Los Angeles, California, and Oak Crest Institute of Science, Pasadena, California, United States of America; University of Oklahoma Health Sciences Center, United States of America

## Abstract

The overwhelming majority of bacteria live in slime embedded microbial communities termed biofilms, which are typically adherent to a surface. However, when several *Staphylococcus epidermidis* strains were cultivated in static liquid cultures, macroscopic aggregates were seen floating within the broth and also sedimented at the test tube bottom. Light- and electron microscopy revealed that early-stage aggregates consisted of bacteria and extracellular matrix, organized in sheet-like structures. Perpendicular under the sheets hung a network of periodically arranged, bacteria-associated strands. During the extended cultivation, the strands of a subpopulation of aggregates developed into cross-connected wall-like structures, in which aligned bacteria formed the walls. The resulting architecture had a compartmentalized appearance. In late-stage cultures, the wall-associated bacteria disintegrated so that, henceforth, the walls were made of the coalescing remnants of lysed bacteria, while the compartment-like organization remained intact. At the same time, the majority of strand-containing aggregates with associated culturable bacteria continued to exist. These observations indicate that some strains of *Staphylococcus epidermidis* are able to build highly sophisticated structures, in which a subpopulation undergoes cell lysis, presumably to provide continued access to nutrients in a nutrient-limited environment, whilst maintaining structural integrity.

## Introduction

In their native habitats, bacteria grow predominantly in multi-cellular communities, which are universally named biofilms [1]. Biofilms are typically found at solid-liquid, solid-gas or liquid-gas interfaces [2], [3]. Nevertheless, most research was done on biofilms at solid-liquid boundaries, which pass through five distinct stages during their development: I) planktonic bacteria, which are suspended in the liquid phase; II) attachment of planktonic bacteria to a solid substrate; III) formation of microcolonies; IV) development into a mature biofilm; V) dispersal of planktonic bacteria from the biofilm [4]. The architecture of mature biofilms varies with the bacterial species and the prevailing physical and physiological conditions. Many reports describe biofilms with an array of mushroom-like towers or with a lawn-like appearance [5], [4]. More infrequent are descriptions of biofilms with a veil- [6], parachute- [7], or honeycomb-like architecture [8]; [9]. *Staphylococcus epidermidis* is a common member of the human skin flora and an important pathogen in nosocomial infections [10]. Whenever *S. epidermidis* is grown at solid-liquid or solid-gas interfaces, they either show a tower or lawn-like biofilm architecture [11], [12]. In static liquid cultures, however, a certain strain of *S. epidermidis* (MH strain), which was isolated from a dog’s canine lymphoma specimen, formed sophisticated structures with “capillary-like networks” or “tissue-like sheets” [13]. When a number of randomly chosen strains (none of them tumor associated) of *S. epidermidis* were tested for their ability to also form the previously observed structures, it became evident that this trade was not an uncommon phenomenon. While the ‘raison d’être’ of these structures is still subject to speculation, the work here presented seeks to reconstruct the chronological development of this unusual biofilm architecture.

## Results

### Early Stage Structures

**Figure 1:**
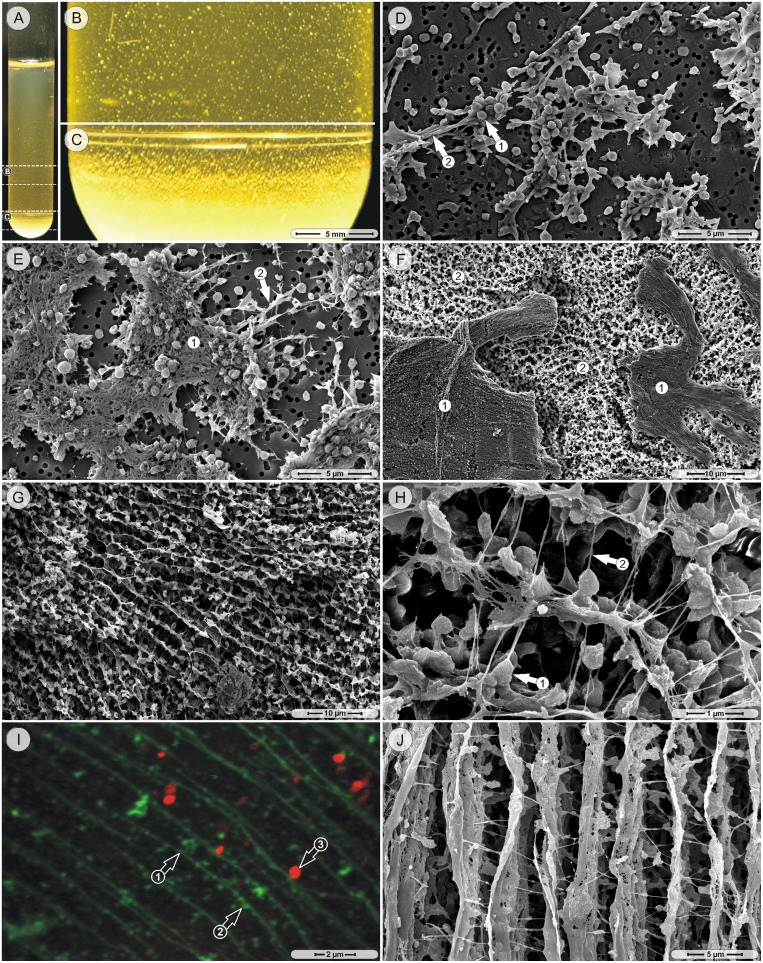
Early stage structures. A–C. Camera pictures, MH strain: floating and sedimented macroscopic aggregates of a 1-day culture. D. Scanning electron microscopy (SEM) picture, MH strain, 1-day culture: bacteria (arrow 1) and matrix elements (arrow 2) form small aggregates. E. SEM picture, 3-day culture, MH strain: a fibrous sheet (region 1) and short strands (arrow 2) form larger aggregates. F. SEM picture, 5-day culture, MH strain: aggregates consist of solid sheets (region 1) and subjacent a network of strands (region 2). G. SEM picture, 5-day culture, MH strain: predominantly parallel orientation of the strand network. H. SEM picture, 5-day culture: bacteria-associated strands (arrow 1), cross-connected by fibers (arrow 2). I. Confocal laser scanning microscopy (cLSM) picture, MH strain, 10-day culture: strands and associated particulate material (arrows 1, 2) are stained with Concanavalin A; bacteria are labeled with Syto59 (arrow 3). J. SEM picture, 10-day culture, MH strain: almost solid, parallel strands with cross-connecting fibers.

Twenty-four hours after the inoculation, floating macroscopic aggregates of all tested strains were visible throughout the liquid volume of the test tube (Fig. 1A–C). Larger aggregates had settled to the bottom of the test tube and formed a sediment (Fig. 1C). Scanning electron microscopic (SEM) examination of floating aggregates (only the MH strain was prepared) from early stage cultures (days 1–3) revealed that they consisted of few aggregated bacteria, which were held together by elements of extracellular matrix (Fig. 1D). In larger aggregates, the extracellular matrix and the bacteria had formed sheet-like structures under which short strands originated and laterally spread (Fig. 1E). Aggregates of 3–5 day old cultures consisted of sheets over a widespread network of strands (Fig. 1F). The strands in this network had a predominantly parallel orientation to each other (Fig. 1G) and were cross-connected with thin fibers (Fig. 1H). The strands themselves were composed of extracellular matrix material and embedded bacteria cells (Fig. 1H). When supernatants of 10-day old cultures were stained with ConA (which binds to *α*-linked mannose and terminal glucose residues), the uniformly aligned strands and the strand-associated particulate material took up this stain, while the nucleic acid specific stain Syto59 labeled the bacteria (Fig. 1I). During the first week of incubation, the number and size of aggregates in the supernatant increased visibly, with larger aggregates settling to the test tube bottom thereby contributing to the progressively enlarging sediment.

### Compartmentalization

**Figure 2:**
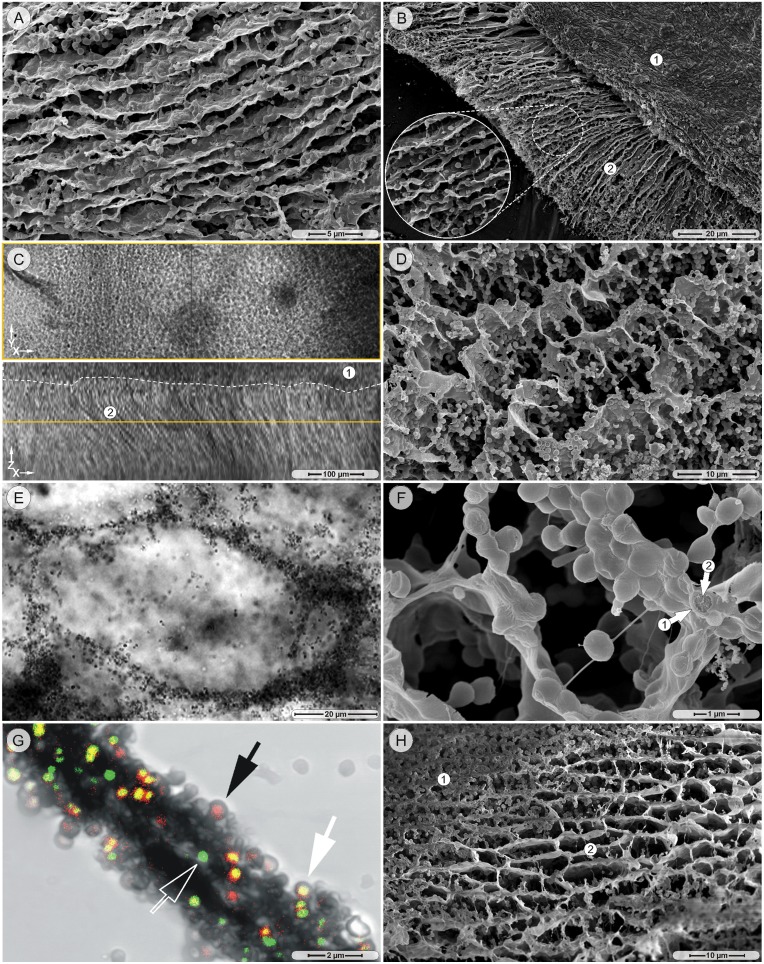
Compartmentalization. A. SEM picture, 10-day culture, MH strain: solid, parallel, wall-like structures. B. SEM picture, 14-day culture, MH strain: the top sheet (region 1) is situated on vertically stacked walls (region 2). C. cLSM transmission mode, 14-day culture, MH strain: a dense, horizontal top plate (region 1) is located on top of vertically stacked walls (region 2). D. SEM picture, 14-day culture, MH strain: compartmentalized structure of aligned walls and cross-walls without top sheet. E. cLSM transmission mode, 14-day culture, MH strain: aligned bacteria form compartment walls. F. SEM picture, 14-day culture, MH strain: matrix (arrow 1) embedded bacteria (arrow 2) form compartment walls. G. cLSM picture, 14-day culture, MH strain: LIVE/DEAD staining of compartment wall forming bacteria show varying stages of membrane integrity (arrows). H. SEM picture, 14-day culture, MH strain: compartment structure with abundant bacteria (region 1) adjacent to an area with bacteria-depleted compartments.

On the tenth day of cultivation, some regions of the aggregates underwent extensive phenotypic changes, while the majority merely continued to grow in size without changing their sheet-strand architecture. During the first stage of this transfiguration, the strands become gradually more solid (Fig. 1J), and turned into compact, but still aligned, wall-like structures (Fig. 2A). Meanwhile, the sheets had become thicker and larger and were found on top of vertically stacked walls (Fig. 2B). When corresponding structures of 14-day old cultures (MH strain) were imaged in the transmission channel of the CLSM, they showed the same general architecture: vertically aligned walls under a noticeably denser and more randomly organized upper-area (Fig. 2C). In regions of the aggregate, where the missing top-sheet allowed an unobstructed view from above onto the overall architecture, a compartmentalized organization with parallel walls and cross-connecting walls became apparent in the SEM (Fig. 2D). Such a structure could extend over large areas of several hundred micrometers. The pronounced compartmentalized architecture was also observed, when walls and cross-walls were visualized under fully hydrated, unfixed conditions in the light microscope (Fig. 2E). A closer examination of the compartment-walls with the SEM showed how the bacteria themselves were aligned as walls (Fig. 2F). A similar pattern of neatly organized bacteria (MH strain) was also observed in the CLSM under unfixed, hydrated conditions (Fig. 2G). Labeling of 14-day old cultures (MH strain) with LIVE/DEAD cell viability staining showed that on average only one third of the cells had intact cell membranes (green staining), while the other two thirds revealed varying stages of compromised cell membranes as evidenced by the yellow-red staining (Fig. 2G).

### Late Stage Structures

**Figure 3:**
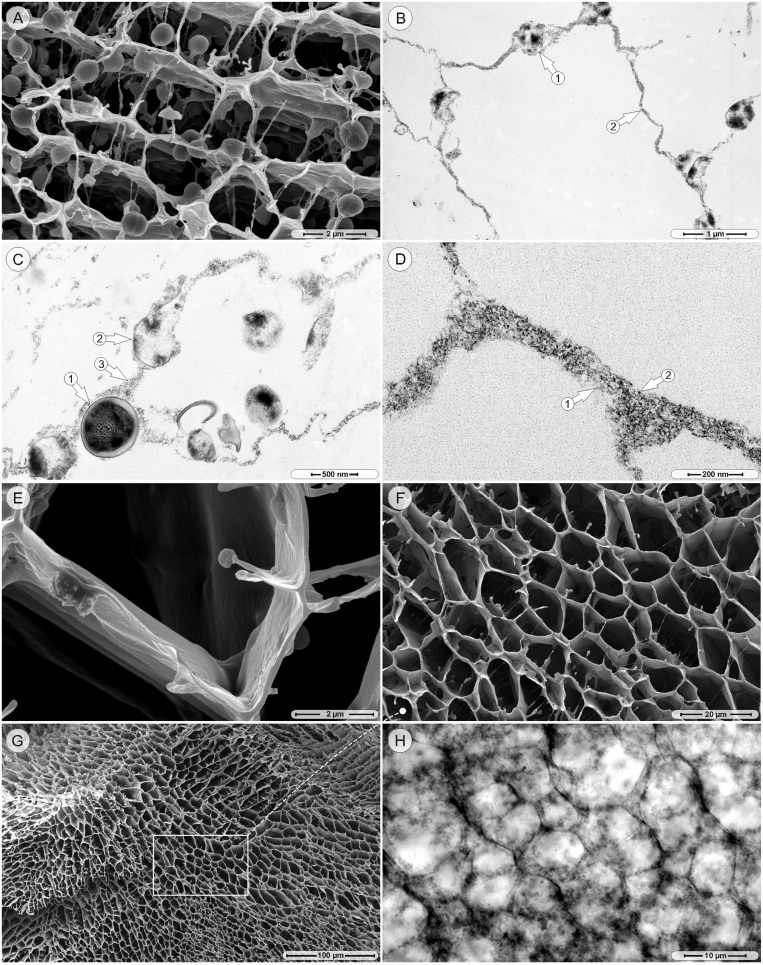
Late stage structures. A. SEM picture, 14-day culture, MH strain: the thin compartment walls contain only few bacteria. B. Transmission electron microscopy (TEM) picture, 14-day culture, MH strain: the few bacteria (arrow 1) are a part of otherwise in ‘empty’ walls (arrow 2). C. TEM picture, 14-day culture, MH strain: an intact bacterium (arrow 1) and a partly disintegrated bacterium (arrow 2) are both integrated in the wall structure (arrow 3). D. TEM picture, 14-day culture, MH strain: the bare walls consist of fine, dispersed fibers (arrow 1) and intensely stained dots (arrow 2). E. SEM picture, 14-day culture, MH strain: empty compartment walls. F. SEM picture, 14-day culture, MH strain: bacteria depleted compartment walls. G. SEM picture: 14-day old cultures (MH strain) showed large compartmentalized areas. H. cLSM transmission mode, 14-day culture (MH strain): ‘empty’ compartment walls under hydrated conditions.

**Figure 4:**
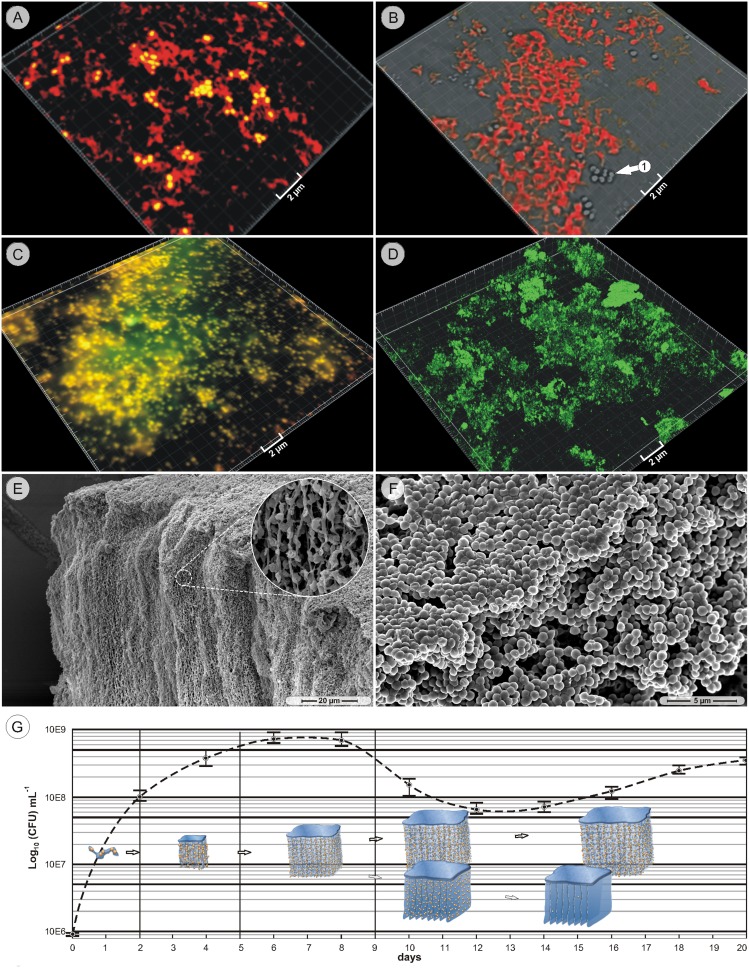
Specific staining, time-course and development model. A. cLSM picture, 14-day culture, strain #49134: the EUB338 probe labels the compartment structure (red), while the bacteria are double-stained by the EUB338 probe and Syto59 (yellow). B. cLSM picture, 14-day culture, MH strain: Concanavalin A stains the compartment structure, but not the bacteria (arrow 1). C. cLSM picture, 14-day culture, strain #49134: the Sypro protein stain (green) and the Syto59 nucleic acid stain label both the bacteria (yellow) but not the compartment structure. D. cLSM picture, 14-day culture, strain #49134: the lipophilic stain Nile Red (green) does not label compartment walls, only diffuse biofilm matrix. E. SEM picture, 28-day culture, MH strain: huge aggregates with intact bacteria and strands continued to be present in long-time cultures. F. SEM picture, MH strain, overnight grown colony on agar. G. The model depicts the main development stages and their timely appearances in the context with the CFU time-course (MH strain).

Commencing on approximately the 12^th^ day of cultivation, several areas of the regular compartmentalized structure contained notably fewer bacteria, while directly adjacent regions still remained unchanged (Fig. 2H). The resulting structure consisted of thin, cross-linked compartment walls with fewer bacteria than previously seen (Fig. 3A), while the majority of the walls were largely devoid of bacteria (Fig. 3B). Only very few bacteria in the walls exhibited still intact cell membranes (Fig. 3C). Most bacteria, however, appeared to be in varying stages of lysis, ranging from partially collapsed cell membranes to completely disintegrated cells with disrupted intracellular morphology (Fig. 3B–C). At higher magnifications, large amounts of dispersed fibers and electron-opaque particles were visible in the compartment walls between the lysing bacteria (Fig. 3D). A SEM examination of bacteria-depleted areas revealed thin walls with no obvious trace of bacteria (Fig. 3E). The overall structure of the now empty compartments still showed the previous organization of walls and cross-walls (Fig. 3F) present over large areas (>500 µm^2^) (Fig. 3G). CLSM imaging of bacteria-depleted compartment walls (MH strain) under unfixed, hydrated conditions confirmed the pattern that was observed by the SEM (Fig. 3H). The concomitant disintegration of cocci, the development of wall-like structures, and the tenor of the TEM pictures (Figs. 3B–D) suggested that the compartment walls were formed from the coalescing remnants of lysed cells. If this assumption was correct, the walls should contain typical intracellular biomolecules such as DNA or ribosomes. To test this hypothesis, specific stains and probes were used. When material from the bottom of the test tube of 14-day old cultures (MH strain) was hybridized with the EUB338 probe, which specifically recognizes a conserved sequence of the 16S rRNA in most bacteria, the compartment-like structures as well as intact bacteria were strongly labeled (Fig. 4A). Additional staining with the DNA specific stain Syto59 in contrast labeled exclusively intact bacteria, while the hybridization with the NONEUB probe (negative control) produced no signal (data not shown). The lectin ConA, with which the strands from the early stages had been successfully stained, also showed affinity to the compartment walls (Fig. 4B), whereas labeling with Wheat Germ Agglutinin (WGA binds to *N*-acetyl glucosamine groups and sialic acid) proved to be inconsistent - only in one out of five times the walls resulted in a signal (data not shown). Co-staining of late-stage cultures with Syto59 and the total protein stain Sypro Orange strongly labeled the cocci, while the space between the bacteria presented only a weak, diffuse signal (Fig. 4C). Labeling with Nile Red, which selectively binds to hydrophobic moieties, produced a signal on the general biofilm matrix, but left the compartment walls unlabeled (Fig. 4D). The attempt to stain possible amyloid structures with Thioflavin T did not result in a signal (data not shown). In summary, strands and compartment walls seemed to consist predominantly of sugars and bacteria, while extracellular DNA, proteins and lipids did not contribute significant amounts. As indicated before, the above-described differentiation concerned only certain regions, while the majority of aggregates increased in size and continued to consist of top-sheets and bacteria-associated strands (Figs. 4E). Although the formation of macroscopic aggregates, strands and compartments was observed in all examined strains, they were more pronounced in cultures of the strains #49134 and MH. The attempt to cultivate late-stage material (28-day old cultures) on agar plates resulted in the formation of colonies with densely arranged cells and interspersed water channels, which are the archetypical features of biofilms at solid-gas interfaces (Fig. 4F). In order to provide a conclusive overview of all structures and their timely appearance, the captured stages were used as raw material to create a basic development model of the aggregates (Fig. 4G).

### CFU Time-course

At the end, a CFU time-course was made to test the assumption, if the observed lysis of a considerable number of bacteria would liberate nutrients and thereby stimulate growth again. The number of bacteria in the time-course peaked around day seven (7.2×10^8^ bacteria mL^−1^), decreased sharply around the tenth day and had a local minimum at the twelfth day of cultivation (1.0×10^8^ bacteria mL^−1^). Between the days 14–18 the number of bacteria recovered again (4.5×10^8^ bacteria mL^−1^) (Fig. 4G).

## Discussion

### Complementary use of Imaging Techniques

The goal of this examination was to reconstruct the developmental stages of an uncommon biofilm structure by *S. epidermidis*. Plunge freezing in liquid propane and freeze substitution, as was employed for all SEM preparations in this study, provides considerably superior structural preservation than chemical fixation [14], although local artifact-formation due to insufficient local freezing is almost unavoidable. In contrast, high pressure freezing in combination with freeze substitution has been proven to be virtually free of artifacts [15], [16]. However, to rule out any possibility that the observed structures (especially late-stage compartments) were artifacts caused by phase separation, complementary light microscopy was also used, since the direct observation of biofilm structures under fully hydrated and unfixed conditions remains the ultimate gold standard [17]. The fact that all main features of the above-described structures were present and had the same arrangement under unfixed and fully hydrated conditions in propane-frozen preparations and with high-pressure processing confirms the reliability of our observations.

### Auto-aggregation

Lotic microbial aggregates have been found in various aquatic systems [3]. Typically, these biofilms form on or adhere to small particles, which are buoyant enough to float. Although floating, the aggregates in the test tube did not adhere to the walls of the test tube, even though *S. epidermidis* is well known to adhere avidly to certain types of plastic, including the polystyrene of the test tubes that was used in this study [18]. Bacterial auto-aggregation enables the development of biofilm by simple cell-to-cell adhesion without the necessity of a solid surface or interface, and is wide spread among oral bacteria and has also been observed in *Yersinia pestis* biofilm formation [19], [20]. In *S. epidermidis* biofilms, intercellular adhesion is commonly mediated by the polysaccharide intercellular adhesin (PIA), and controlled by the *icaADBC* operon [21], [22]. The strains in this study included three *ica*-negative strains (#12228, #14990 and #49134), the strain #35547 was positive for ica A, icaC’ and ica R [23], whereas the ica-status of the MH-strain was not established. Since all strains showed auto-aggregation and the development of aggregates, their formation cannot be based on PIA alone. Instead of PIA, *ica-*negative *S. epidermidis* strains appear to use a number of proteins (e.g. Embp, Aap and Bhp) for auto-aggregation and biofilm formation [24]. A strong hint for the involvement of protein in the auto-aggregation was delivered during the attempt to embed chemically fixed aggregates for TEM. After the specimen was stained with 1% osmium tetroxide at room temperature the entire structure broke apart within minutes into little fragments. The proteolytic properties of osmium tetroxide have been described previously [25]. The above-described observation would be consistent with a model, in which proteins are associated with auto-aggregation and the integrity of aggregates.

### Aggregate Assembly

As the term ‘auto’-aggregation implies, the first stages in aggregate formation are probably based on the binding of specific proteins on the bacterial surface. But only if the resulting sheet-aggregate were assumed to float, the associated strands would be able develop underneath of such a structure. The parallel alignment of the strands in static liquid media seems to have one obvious source, gravitation force, along which the strands apparently develop and prolong, as indicated in the model in Fig. 4G.

### Subpopulation, Mass Transfer and Cell Death

Between the seventh and tenth day of cultivation a subpopulation of bacteria-associated strands transformed into compartment walls, and eventually underwent cell lysis. Phenotypic variations of a subpopulation have been reported at frequencies between 10^−3^ and 10^−4^, although to date only for *ica*-positive *S. epidermidis* strains, when they were cultivated for an extended period (5–7 days) [26]. Similarly, programmed cell death in biofilms is also known to only affect a subpopulation of a bacterial community [27], [28]. So far, several reasons for this phenomenon have been discussed, two of which seem suitable to shed some light on the scenario observed in this study. At first, the numeric reduction of competing individuals in a stage of nutrient limitation is posited to release the pressure on nutritive substances; and second, bacterial cell lysis is hypothesized to liberate fresh nutrients in a nutrient-limited situation [29], [28], The numeric reduction of bacteria numbers in the CFU time-course coincides with the formation of compartment walls and bacterial lysis, while the concomitant appearance of walls without intact bacteria and the slow rise in CFU counts could hint at a putative release and recycling of nutrients. In contrast to the classic biofilm architecture with comparatively densely packed cells, the strand network as well as the compartment structure has a more “open space” architecture, which implies a significantly better mass transfer between nutrients and bacteria [30]. In view of the static conditions in the test tube it is likely that the late-stage compartment walls serve, indeed, as a nutrients source with advantageous mass transfer properties.

### Specific Staining

The strands and the compartment walls evolving from the strands revealed both a strong affinity for the ConA lectin, which underlines their close structural ties. ConA specifically binds to α-linked mannose and terminal glucose residues and has been used to type *S. epidermidis* strains [31]. Whereas α-mannose has never been detected in *S. epidermidis* biofilms, 1,6-β-D- glucosaminoglycan is a well-known component of their biofilm matrix [32]. However, only *ica*-positive strains should have the genetic traits to produce this sugar polymer. The origin of the glucose residues in the strands and in the compartment walls is therefore not clear. The bacterial cytoplasm contains larger amounts of proteins and nucleic acids, which were released during the observed cell lysis according to the TEM pictures, and formed the late-stage compartment walls. Consequently, one would expect to find detectable amounts of cytoplasmic proteins and nucleic acids in these structures. But neither strands nor walls were labeled with stains for proteins, lipids or DNA, only the FISH probe was able to detect ribosomes in the compartment walls. It is conceivable that the denaturating conditions of the fluorescence *in situ* hybridization process created the access for the probes to the target, while in non-denaturating situations the target moieties in the walls are masked.

### In Search of a Habitat

Our observations raise an interesting question: in which natural habitats are *S. epidermidis* likely to form such structures? It is difficult to imagine that *S. epidermidis* can take advantage of its aggregate-forming abilities in its classic habitats, since human skin, the nasal mucus membrane, food, and polymer surfaces are mostly environments at solid-gas interphase or at solid-liquid-film interphases at best. Nevertheless, Staphylococci are also known for blood stream infections [10]. The laminar flow in this habitat could offer the physical preconditions for the formation of aggregates with cross-connected long strands. Analogous structures are formed by neutrophils, which function as filters to capture microbial pathogens [33]. It is possible that the strands might provide a similar protection of the bacteria from host defenses. However, teleological driven considerations suggest a habitat with static or low flow liquid conditions, as might be found in an abscess, where the possession of an appropriate set of genes are of advantage for the strains to survive periods with limited access to nutrients, whilst still maintaining structural integrity.

## Materials and Methods

### Strains

All strains of *S. epidermidis* (ATCC #14490, #12228, #35547 and #49134) were obtained from the American Type Culture Collection (Manassas, VA, USA), except the “MH” strain, which was isolated from a canine lymphoma as described previously [13]. The strains #14490, #12228, and #35547 were only prepared for light microscopic examination and served as confirmation that the observed structures were not restricted to a very limited number of *S. epidermidis* strains.

### Static Cultivation in Liquid Media

15 mL plastic test tubes (polystyrene) were filled with 7 mL trypticase soy broth (Thermo Fisher Scientific Remel Products, Lenexa, KS, USA), inoculated from an overnight agar plate with a single *S. epidermidis* strain, vortexed for 30 sec and incubated at 37°C without shaking or media change.

### CFU Time-course

Triplicates of test tubes cultures (MH strain) were cultivated for 0, 2, 4, 6, 8, 10, 12, 14, 16 and 18 days as described above. For the CFU determination, the cultures were vortexed for 30 sec, diluted appropriately, plated on tryptic soy agar plates and incubated over night. The CFUs were counted and the median determined.

### Cultivation on Agar

Sterile Isopore™ membrane filters (0.4 µm, HTTP, Millipore, Ireland) were placed on tryptic soy agar plates (Thermo Fisher Scientific Remel Products, Lenexa, KS, USA). The test tube of 28-day old static cultures (MH strain) was vigorously vortexed for 30 sec, 50 µL were spot deposited on each filter, spread with a spatula and incubated for one day at 37°C. The filters were then prepared for SEM imaging as described below.

### Macroscopic Imaging

The development of the macroscopic structures (MH strain) in the test tubes was documented under back lighting conditions with a digital camera and a macro lens (Nikon D70s, Nikon, Inc. Melville, NY, USA. Sigma 28–300 mm F3.5–6.3 Macro, Sigma Corp. Ronkonkoma, NY, USA).

### Specific Staining and Confocal Laser Scanning Microscopy (CLSM)

Material from the upper test tube volume and the test tube bottom of 14-day old cultures (#49134 and MH strain) was removed by gentle suction using a cut-off 1 mL pipette tips (to minimize shearing), and labeled with LIVE/DEAD BacLight (Invitrogen, Carlsbad, CA, USA) according to the manufactures instructions. In the same way, more 14-day old cultures were stained with Concanavalin A (25 µg mL^−1^, (ConA, Vector Laboratories, Inc., Burlingame, CA, USA) and Syto59 (5 µM, Invitrogen, Carlsbad, CA, USA), or Wheat Germ Agglutinin (25 µg mL^−1^) (Vector Laboratories, Inc.) and Syto59, or Sypro Orange and Syto59, or Nile Red (5 µg mL^−1^, Sigma-Aldrich Corp. St. Louis, MO, USA), or Thioflavin T (20 mM, Sigma-Aldrich). Fluorescence *in situ* hybridization (FISH) was performed on material collected from the test tube bottom, which was hybridized with the EUB338-Cy3 probe [34] or the NONEUB-Cy5 probe [35] at a final concentration of 5 ng µL^−1^ for 90 min at 46°C. These specimens were counter-stained with Syto59. All samples were examined either with a Leica DM RXE microscope with a TCS SP2 AOBS confocal system (Leica Microsystem, Exton, PA, USA) or a LSM710 (Carl Zeiss MicroImaging, Inc., Thornwood, NY, USA.) using confocal and transmitted imaging with the appropriate laser wavelengths lines and detection windows.

### Time-course for SEM

*S. epidermidis* cultures (MH strain) were cultivated for of 1, 2, 3, 5, 7, 10, 14 and 28-days as described above. 100 µL from the sedimented material at the test tube bottom was removed by gentle suction using cut-off 1 mL pipette tips, spot deposited on a round glass cover slip (12 mm) and rapidly frozen by immersion in liquid propane. The same protocol was repeated with 100 µL media of the supernatant. For freeze substitution, all specimens were quickly transferred, (still frozen) to pre-cooled vials containing 100% ethanol, which were placed in a Styrofoam container with dry ice. Subsequently, the container was left at −20°C overnight and then warmed to 4°C over a period of 8 h. Afterwards, the specimens were critical point dried, mounted on a stub with adhesive carbon tape, sputter coated with a 25 nm layer of platinum and examined in the SEM operating at 5 kV in the secondary electron mode (XL 30 S, FEG, FEI Company, Hillsboro, OR, USA).

### Failed Attempt to Embed Chemically Fixated Aggregates

*S. epidermidis* (MH strain) was cultivated for 14 days as described above. Material from the bottom of the test tube was chemically fixed (2.5% glutaraldehyde, 4% paraformaldehyde in 50 mM HEPES buffer) for 48 h, stained with 1% osmium tetroxide at room temperature. Since the aggregates disassembled into small fragments during this step the embedding was not further processed.

### Cryo-fixation, Freeze-substitution and TEM

Autoclaved 1 mm segments of Spectra/Por *in vivo* microdialysis hollow fibers (MWCO 13 kD, Spectrum Laboratories, Inc. Rancho Dominguez, CA, USA) were incubated in *S. epidermidis* (MH strain) cultures as described above for 14 days. The micro-dialysis tubes at the bottom of the test tube were removed, their ends crimped shut and placed in aluminum planchettes, in which the remaining space was filled with a 20% (w/v) bovine serum albumin buffered solution. The samples were high pressure frozen (Bal-Tec HPM-010, Bal-Tec, Inc., Carlsbad, CA, USA), held at liquid nitrogen temperature after freezing and then gradually warmed to −90°C over a period of 16 h in an ASF2 (Leica Microsystems, Inc., Deerfield, IL, USA) in the presence of 100% ethanol containing 1% osmium tetroxide. Subsequently, the specimens were warmed to −60°C over a 24 h period and held for another 24 h in fresh 100% ethanol (without osmium tetroxide) with three changes of ethanol. All specimens were finally warmed to ambient temperature (22°C) overnight and embedded in epoxy resin (Epon 812 substitute, EMS Hadfield, PA, USA). Thin sections (50–70 nm) were prepared using an Ultracut UC6 (Leica Microsystems, Inc., Deerfield, IL, USA) and post-stained with 1% aqueous uranyl acetate and Reynold’s lead citrate for examination by transmission electron microscopy operating at 80 kV (FEI CM120, FEI Inc., Hillsboro, OR, USA). TEM images were collected in negative film and digitally scanned.

### Image Processing

Images have been cropped and adjusted for optimal brightness and contrast (applied to the whole image) using Photoshop (Adobe Systems, San Jose, CA, USA).
